# Biological evaluation and structure activity relationship of 9-methyl-1-phenyl-*9H*-pyrido[3,4-*b*]indole derivatives as anti-leishmanial agents

**DOI:** 10.1016/j.bioorg.2018.11.037

**Published:** 2019-03

**Authors:** Penta Ashok, Subhash Chander, Terry K. Smith, Rajnish Prakash Singh, Prabhat Nath Jha, Murugesan Sankaranarayanan

**Affiliations:** a Medicinal Chemistry Research Laboratory, Department of Pharmacy, Birla Institute of Technology & Science Pilani, Pilani Campus, Pilani 333031, Rajasthan, India; b School of Pharmacy, Maharaja Agrasen University, Atal ShikshaKunj, Solan, Himachal Pradesh 174103, India; c Schools of Biology & Chemistry, BSRC, The University, St. Andrews, Fife Scotland. KY16 9ST, UK; d Department of Biological Sciences, Birla Institute of Technology & Science Pilani, Pilani Campus, Pilani 333031, Rajasthan, India

**Keywords:** Molecular hybridization, Leishmaniasis, *Leishmania infantum*, *Leishmania donovani*, Promastigotes, Amastigotes

## Abstract

A series of piperazinyl-β-carboline-3-carboxamide derivatives were designed through a molecular hybridization approach. Designed analogues were synthesized, characterized and evaluated for anti-leishmanial activity against *Leishmania infantum* and *Leishmania donovani*. In *L. infantum* inhibition assay*,* compounds **7d**, **7g** and **7c** displayed potent inhibition of promastigotes (EC_50_ 1.59, 1.47 and 3.73 µM respectively) and amastigotes (EC_50_ 1.4, 1.9 and 2.6 µM respectively). SAR studies revealed that, para substitution of methoxy, chloro groups and methyl group on ortho position favored anti-leishmanial activity against *L. infantum*. Among these analogues **7d**, **7h, 7n** and **7g** exhibited potent inhibition against *L. donovani* promastigotes (EC_50_ 0.91, 4.0, 4.57 and 5.02 µM respectively), axenic amastigotes (EC_50_ 0.9, 3.5, 2.2 and 3.8 µM respectively) and intracellular amastigotes (EC_50_ 1.3, 7.8, 5.6 and 6.3 µM respectively). SAR studies suggested that, para substitution of methoxy group, para and meta substitution of chloro groups and benzyl replacement recommended for significant anti-leishmanial against *L. donovani*.

## Highlights

• New anti-leishmanial agents designed through molecular hybridization approach.
• 7d showed potent anti-leishmanial activity against both *L. infantum* & *L. donovani.*
• 7d EC_50_ against *L. infantum* promastigotes, axenic amastigotes 1.59 & 1.4 µM.
• 7d EC_50_ against *L. donovani* promastigotes, axenic & intracellular amastigotes 0.91 & 0.91 & 1.4 µM.

## Graphical abstract

Anti-leishmanial activity EC_50_ (µM).

*L. infantum:* promastigotes 1.59 and axenic amastigotes 1.4.

*L. donovani:* promastigotes 0.9, axenic amastigotes 1.4 and intracellular amstigotes 1.3.

**Figure:**
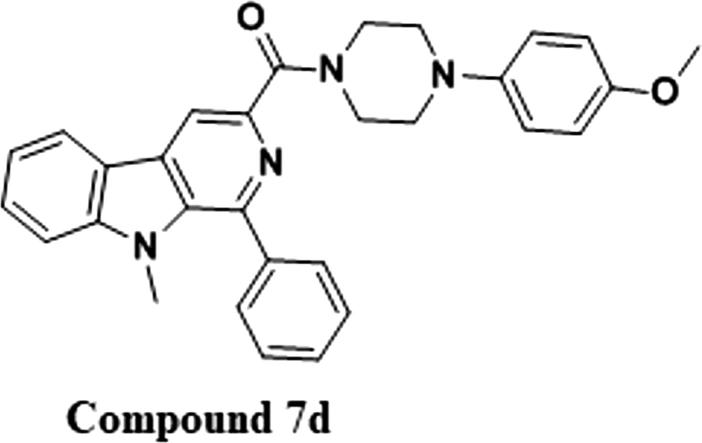

## 1 Introduction

Leishmaniasis is caused by intracellular protozoan *Leishmania* spp parasites and is considered as one of the most neglected tropical diseases. Among various clinical forms of leishmaniasis, visceral leishmaniasis is the most severe, affecting internal organs like bone marrow, liver and spleen. VL is also known as kala-azar and is lethal if untreated in over 95% of cases. VL caused by *Leishmania donovani* and *Leishmania infantum* [1], [2]. Leishmaniasis control is majorly dependent upon chemotherapy that includes decade old drugs, due to no effective vaccines. Antimonial drugs such as sodium stibogluconate and meglumineantimoniate have been used for the last 70 years, however increasing incidence of resistance has been reported [3]. Anti-leishmanial drugs such as amphotericin B, diamidine, pentamidine and paromomycin has been restricted by cost, toxicity and resistance [4], [5]. Miltefosine is originally developed as anti-cancer agent, has been approved by FDA for leishmaniasis treatment [6]. Miltefosine has restricted use in pregnancy and has high risk of resistance due to long half-life (150 h). Clinical efficacy of miltefosine has been decreased in countries like India, where it is used extensively [7]. Based upon these collective facts, there is an increased need of new anti-leishmanial agents with good therapeutic profiles.

**Fig. 1:**
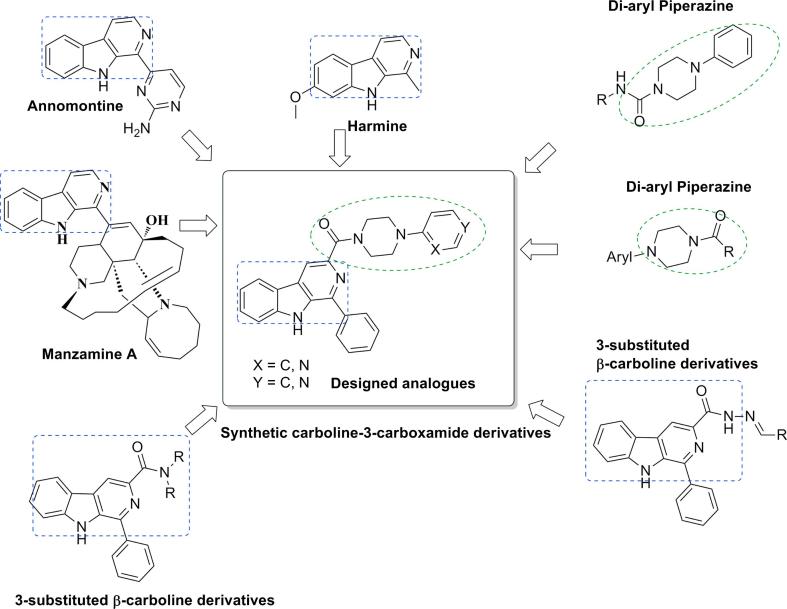
Structure of reported and designed β-carboline-piperazine hybrid molecules.

Nature and natural products remain an important source for the development of therapeutic agents. Natural products especially alkaloids have been known for anti-infective activity. β-carboline represents a tricyclic pyrido[3,4-*b*]indole ring system has reported for wide spectrum of biological activities. β-carboline derivatives displayed different biological activities such as anti-microbial [8], anti-cancer [9], anti-leishmanial [10], [11], anti-malarial [12], [13], anti-tubercular [14], anti-viral [15] and anti-thrombotic activities [16]. β-carboline alkaloids such as Harmine, annomontine and manzamines (Fig. 1) exhibited potent anti-leishmanial activity [10], [17], [18]. Manzamine is a group of β-carboline alkaloids, possess complex structure of β-carboline moiety attached to a pentacyclic diamine ring having both eight and thirteen membered rings on a pyrrolo[2,3-*i*] isoquinoline framework. Large numbers of manzamine alkaloids are isolated from different marine sponge species and exhibited potent anti-leishmanial activity. Beside these natural β-carboline alkaloids, several synthetic β-carboline derivatives having different substitutions on 1, 2, 3 and 9 positions have exhibited significant anti-leishmanial activity [18], [19]. Among these synthetic β-carboline derivatives, β-carboline derivatives with *N*-alkylcarboxamide group on 3 position displayed potent anti-leishmanial activity and attracted us to design new β-carboline derivatives [20], [21], [22], [23]. The piperazine moiety has privileged position in medicinal chemistry with diverse biological activities [24]. Bisarylpiperazine derivatives exhibited activities such as anti-leishmanial [25], anti-microbial [24], [26], [27], anti-protozoal [28], [29], anti-cancer [30], anti-tubercular [30], [31] and anti-viral [32] (Fig. 1). Based upon literature reports, in the present study we designed β-carboline and piperazine hybrid molecules (Fig. 1) using molecular hybridization approach.

## 2 Results and discussion

### 2.1 Chemistry

**Scheme 1:**
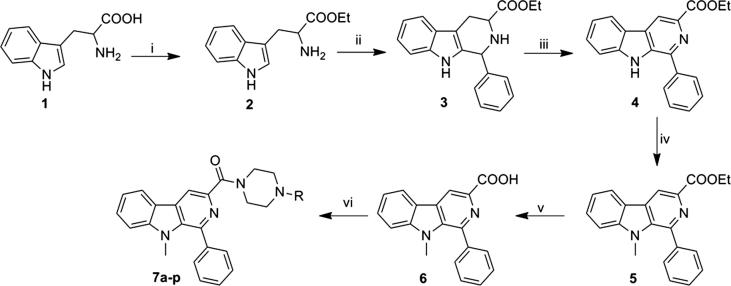
Reagents and conditions: (i) thionylchloride, ethanol, reflux, 30 min, 76%; (ii) benzaldehyde, trifluoroacetic acid, DCM, rt, 3 h, 82%; (iii) KMnO_4_, THF, rt, 24 h, 68%; (iv) methyliodide, KOH, DMSO, rt, 30 min, 72%; (v) 50% aq. NaOH, reflux, 30 min, 78%; (vi) EDCl, HOBt, THF, piperazines, 0 °C-rt 6 h, 62–82%.

The synthetic scheme for β-carbolinepiperazine derivatives was illustrated in Scheme 1. The titled compounds were synthesized using DL-Tryptophan **(1)** as starting material. Initial esterification of DL-Tryptophan **(1)** using thionylchloride to get an ethyl ester of tryptophan **(2)**, was followed by pictet-spengler reaction in presence of trifluoroacetic acid to obtain tricyclic ethyl 2,3,4,9-tetrahydro-1-phenyl-1*H*-pyrido[3,4-*b*]indole-3-carboxylate **(3).** Upon oxidation with potassium permanganate, ethyl-1-phenyl-9*H*-pyrido[3,4-*b*]indole-3-carboxylate **(4)** was obtained, and is continued by 9-*N* methylation with methyl iodide in presence of potassium hydroxide to acquire ethyl-9-methyl-1-phenyl-9*H*-pyrido[3,4-*b*]indole-3-carboxylate **(5)**. Further alkaline ester hydrolysis of compound **(5)** produced 9-methyl-1-phenyl-9*H*-pyrido[3,4-*b*]indole-3-carboxylic acid **(6)** as key intermediate. The carboxylic acid key intermediate **(6)** was treated with appropriate amines (aryl-substitutedpiperazines) in presence of coupling agent 1-ethyl-3-(3-dimethylaminopropyl)carbodiimide hydrochloride (EDCI) and hydroxybenzotriazole (HOBt) to obtain the desired products **(7a-p)** in good yields [33], [34], [35].

### 2.2 Biological evaluation

#### 2.2.1 Cytotoxicity evaluation

Initially all these reported β-carboline derivatives were evaluated for cytotoxicity against HeLa cell lines at 500 μM by an Alamar blue assay, which showed most of these compounds were non-toxic to HeLa cells at tested concentration. Compounds which showed cytotoxicity against HeLa cells were further evaluated to determine cytotoxic concentration (CC_50_) value. Among these reported β-carboline derivatives, compound **7o** and **7p** showed cytotoxicity (CC_50_ 29.7 and 27.3 μM, respectively) against HeLa cells.

#### 2.2.2 Anti-leishmanial screening

Anti-leishmanial activity of these analogues was determined by evaluating their inhibition activity against both promastigote, amastigote forms of *Leishmania infantum* and *Leishmania donovani* strains. Compounds were screened against promastigote forms of the *Leishmania* strains to determine their effective concentration (EC_50_) values. Finally, compounds which exhibited potent inhibition of promastigote (EC_50_ < 20 µM) were further evaluated against amastigote forms of respective strain using established axenic and intracellular amastigote assay methodologies. Anti-leishmanial drugs miltefosine and pentamidine were used as standards for comparison purpose.

##### 2.2.2.1 Leishmaniainfantum

###### 2.2.2.1.1 Anti-promastigote activity

Anti-promastigote activity of these reported β-carboline derivatives was determined by Alamar blue assay method. In *in-vitro* evaluation against promastigotes of *L. infantum,* compounds **7g, 7d,** and **7c** displayed potent anti-promastigote activity (EC_50_ 1.47, 1.59 and 3.73 µM respectively) than standard drugs pentamidine and miltefosine (EC_50_ 8.31 and 12.36 µM respectively). Compounds **7a, 7f, 7j** and **7k** displayed significant inhibition of *L. infantum* promastigotes (EC_50_ 9.45, 13.8, 19.7 and 17.2 µM respectively) and inhibition potency was comparable with standard drugs. Analogues **7n**, **7b** and **7h** exhibited moderate activity (EC_50_ 20.4, 24.3 and 21.7 µM respectively), while **7d**, **7h**, **7l**, **7m**, **7o** and **7p** showed weak anti-promastigote activity (EC_50_ 72.4, 50.9, 64.9, 93.0, 29.7 and 45.7 µM respectively) against *L. infantum*. Especially, compounds **7d** and **7g** exhibited most promising inhibition of *L. infantum* and are five times more potent than the standard drugs. Structure activity relationship studies suggested that, para substitution with ortho-para directing groups (methoxy and chloro) enhanced promastigotes inhibition activity significantly. Ortho substitution with methyl group has increased the anti-promastigote activity significantly while substitution of other ortho-para directing and meta directing groups (methoxy, chloro) substitution on phenyl ring has decreased the activity drastically. Replacement of phenyl ring with benzyl and pyridyl ring was not favorable for anti-promastigote activity.

###### 2.2.2.1.2 Anti-amastigote activity

Compounds exhibited significant inhibition of *L. infantum* promastigotes (EC_50_ < 20 μM) were further evaluated against axenic amastigote forms of *L. infantum.* Alamar blue assay method was used to determine inhibition potential of these β-carboline derivatives against (human life stage forms) axenic amastigotes. In *L. infantum* axenic amastigote inhibition assay, compounds **7d, 7g** and **7c** displayed potent activity (EC_50_ 1.4, 1.9 and 2.6 µM respectively) better than standard drugs pentamidine and miltefosine (EC_50_ 2.7 and 4.8 µM). Compound **7a** showed axenic anti-amastigote activity (EC_50_ 4.5 µM) against *L. infantum* and potency was comparable with standard drugs. Compounds, **7j**, **7k** and **7f** exhibited significant inhibition of *L. infantum* axenic amastigotes (EC_50_ 14.6, 15.0 and 15.3). *In-vitro* anti-amastigote activity results (Table 1) suggested that these β-carboline derivatives were more potent against axenic amastigote forms of *L. infantum*.

**Table 1:** Anti-leishmanial activity of the titled compounds against *L. infantum.*

Comp. Code	R	CC_50_ (µM)	*L. infantum* promastigote EC_50_ (µM)	S.I^a^	*L. infantum Axenic* amastigote EC_50_ (µM)	S.I^a^
**7a**	–C_6_H_5_	>500	9.45 ± 0.82	>52.9	4.5 ± 0.3	>111.1
**7b**	–4CH_3_C_6_H_4_	>500	24.3 ± 1.3	>20.5	ND	–
**7c**	–2CH_3_C_6_H_4_	>500	3.73 ± 0.28	>134.0	2.6 ± 0.2	>192.3
**7d**	–4OCH_3_C_6_H_4_	>500	1.59 ± 0.11	>314.5	1.4 ± 0.1	>357.1
**7e**	–3OCH_3_C_6_H_4_	>500	72.4 ± 6.7	>6.9	ND	–
**7f**	–2OCH_3_C_6_H_4_	>500	13.8 ± 0.9	>36.2	15.3 ± 1.1	>32.7
**7g**	–4ClC_6_H_4_	>500	1.47 ± 0.32	>340.1	1.9 ± 0.2	>263.2
**7h**	–3ClC_6_H_4_	>500	50.9 ± 4.8	>9.8	ND	–
**7i**	–2ClC_6_H_4_	>500	21.7 ± 0.6	>23.0	ND	–
**7j**	–4NO_2_C_6_H_4_	>500	19.7 ± 1.7	>25.3	14.6 ± 2.9	>34.2
**7k**	–4FC_6_H_4_	>500	17.2 ± 0.82	>29.1	15.0 ± 1.3	>33.3
**7l**	–2FC_6_H_4_	>500	64.9 ± 5.7	>7.7	ND	–
**7m**	–2,3-diClC_6_H_5_	>500	93.0 ± 7.5	>5.3	ND	–
**7n**	–CH_2_C_6_H_5_	>500	20.4 ± 1.4	>24.5	ND	–
**7o**	–4C_5_H_4_N	29.7 ± 1.5	61.1 ± 5.4	0.48	ND	–
**7p**	–2C_5_H_4_N	273 ± 21.6	45.7 ± 3.3	5.97	ND	–
Pentamidine			8.31 ± 0.18		2.7 ± 0.4	
Miltefosine			12.6 ± 1.1		4.8 ± 0.8	

a SI = CC_50_/IC_50,_ ND – Not Determined.

*Structure Activity Relationship (SAR).* Among these piperzinyl-β-carboline derivatives, un-substituted proto compound displayed significant anti-leishmanial activity against both promastigote and amastigotes of *L. infantum*. A series of β-carboline derivatives with different ortho-para and meta directing groups on various positions of phenyl were studied to determine their effect on anti-leishmanial activity. Substitution of ortho-para directing methyl group on ortho position of phenyl ring has increased anti-leishmanial activity significantly against both forms, whereas methyl substitution on para position has reduced the activity. Ortho-para directing groups such as methoxy and chloro on para substitution strongly enhanced the anti-leishmanial activity, while ortho substitution lessen the activity and meta substitution drastically decreased the activity. Meta directing nitro and fluoro group substitution on para position decreased the activity considerably and fluoro substitution on ortho position severely decreased the activity. Dual substitution on ortho and meta position with chloro declined the activity. Moreover, phenyl ring replacement with pyridine and benzyl ring has adversely affected the anti-leishmanial activity. SAR studies suggested that, meta position of phenyl ring is highly susceptible, substitution with any group drastically reduced the activity. Ortho-para directing methoxy, chloro groups on para position and methyl group on ortho position favored the activity. Phenyl ring replacement with heterocyclic rings is not recommended for anti-leishmanial activity against *L. infantum*.

##### 2.2.2.2 Leishmaniadonovani

###### 2.2.2.2.1 Anti-promastigote activity

*L. donovani* promastigote inhibition potency of these reported β-carboline derivatives was determined by Alamar blue assay method. Among these analogues, compound **7d** exhibited potent inhibition (EC_50_ 0.91 µM) of *L. donovani* promastigotes than standard drugs pentamidine and miltefosine (EC_50_ 6.4 and 3.12 µM). Anti-promastigote activity of analogues **7h, 7n** and **7g** (EC_50_ 4.0, 4.57, and 5.02 µM respectively) is equipotent as that of standard drugs. Compounds **7f**, **7p**, **7c**, **7a**, **7k**, **7j**, **7b**, **7i**, and **7o**displayedsignificant inhibition (EC_50_ 8.42, 8.5, 9.45, 11.9, 12.4, 13.1, 14.2, 15.2 and 19.5 µM respectively) of promastigote and inhibition potency is comparable with standard drugs. Compound **7e** showed moderate (EC_50_ 28.5 µM) anti-promastigote activity and **7l** and **7m** displayed weak inhibition (EC_50_ 85.9 and 117.5 µM) of *L. donovani* promastigotes.

###### 2.2.2.2.2 Axenic amastigote screening

Compounds exhibited significant anti-promastigote activity (EC_50_ < 20 µM), were further screened against axenic amastigote (human life cycle parasite form) forms. Among these derivatives, Analogues **7d**, **7h, 7n** and **7g** showed potent inhibition (EC_50_ 0.9, 2.2 3.5 and 3.8 µM respectively) of axenic amastigotes. Especially, compound **7d** exhibited two to three times more potent anti-amastigote activity than pentamidine and miltefosine (EC_50_ 1.6 and 2.8 µM). Compounds **7c**, **7b**, **7f**, **7j**, **7k** and **7a** exhibited significant inhibition (EC_50_ 5.3, 5.8, 6.4, 7.5, 9.3 and 10.0 µM respectively) of *L. donovani* axenic amastigotes. Compounds **7p**, **7o** and **7i** displayed moderate anti-amastigote activity (EC_50_ 12.3, 13.9 and 19.4 µM respectively). Axenic amastigote assay results revealed that these β-carboline derivatives exhibited potent inhibition of axenic amastigotes of *L. donovani.*

###### 2.2.2.2.3 Intracellular amastigote screening

Although, axenic amastigotes assay most commonly used to screen new leishmanial agents are different from intracellular amastigotes in terms of protein expression and drug susceptibility. Hence in our present study, compounds displayed potent inhibition of promastigote and axenic amastigotes were further evaluated against intracellular amastigotes to ensure their activity against clinical relevant forms of parasite. Intracellular amastigote assay results suggested that, most of these analogues displayed potent inhibition of *L. donovani*macrophagic amastigotesand the potency was comparable with miltefosine and many folds higher than pentamidine (Table 2). Especially, compound **7d** ((4-(4-methoxyphenyl)piperazin-1-yl)(9-methyl-1-phenyl-9*H*-pyrido[3,4-*b*]indol-3-yl)methan-one) exhibited five times potent inhibition (EC_50_ 1.3 µM) of intracellular amastigotes compared to miltefosine (EC_50_ 6.4 µM). Compounds **7n** and **7g** were displayed potent anti-amastigote activity (EC_50_ 5.6 and 6.3 µM) than standard drugs miltefosine and pentamidine with better selectivity index. Anti-amastigote potency of **7b**, **7c**, **7h, 7o** and **7p** (EC_50_ 7.2, 7.8, 7.9, 7.9 and 9.4 µM) is comparable with miltefosine and better than pentamidine. Compounds **7k, 7f**, **7a**, **7j** and **7i** exhibited good anti-amastigote activity (EC_50_ 10.1, 11.8, 13.5, 15.4 and 22.0 µM) with better selectivity.

**Table 2:** Anti-leishmanial activity of the titled compounds against *L. donovani.*

Compound Code	CC_50_ (µM)	*L. donovani Promastigote* EC_50_ (µM)	S.I^a^	*L. donovani Axenic amastigote* EC_50_ (µM)	S.I^a^	*L. donovani intracellular a*mastigote EC_50_ (µM)	S.I^a^
**7a**	>500	11.9 ± 1.2	>42.0	10.0 ± 0.3	50.0	13.5 ± 2.0	37.0
**7b**	>500	14.2 ± 1.2	>35.2	5.8 ± 0.3	86.2	7.9 ± 0.4	63.3
**7c**	>500	9.45 ± 0.72	>52.9	5.3 ± 0.4	94.3	9.4 ± 1.3	53.2
**7d**	>500	0.91 ± 0.09	>549.5	0.9 ± 0.1	555.6	1.3 ± 0.1	384.6
**7e**	>500	28.5 ± 1.8	>17.5	ND	–	ND	–
**7f**	>500	8.42 ± 0.45	>59.4	6.4 ± 0.2	78.1	11.8 ± 0.7	42.4
**7g**	>500	5.02 ± 0.36	>99.6	3.8 ± 0.5	131.6	6.3 ± 0.3	79.4
**7h**	>500	4.0 ± 0.25	>125	2.2 ± 0.1	227.3	7.8 ± 1.3	64.1
**7i**	>500	15.2 ± 0.85	>32.9	19.4 ± 1.2	25.8	22.0 ± 2.1	22.7
**7j**	>500	13.1 ± 1.5	>38.2	7.5 ± 0.4	66.7	15.4 ± 0.9	32.5
**7k**	>500	12.4 ± 1.4	>40.3	9.3 ± 0.5	53.8	10.1 ± 1.1	49.5
**7l**	>500	85.9 ± 7.3	>5.8	ND	–	ND	–
**7m**	>500	117.5 ± 6.4	>4.3	ND	–	ND	–
**7n**	>500	4.57 ± 0.24	>109.4	3.5 ± 0.2	142.9	5.6 ± 0.9	89.3
**7o**	29.7 ± 1.5	19.5 ± 2.8	1.52	13.9 ± 0.4	2.1	7.2 ± 0.5	4.1
**7p**	273 ± 21.6	8.5 ± 0.75	45.73	12.3 ± 0.6	22.2	7.9 ± 0.3	34.6
Pentamidine		6.40 ± 0.11		1.6 ± 0.1		23.7 ± 1.8	
Miltefosine		3.12 ± 0.16		2.8 ± 0.4		6.4 ± 0.3	

a SI = CC_50_/IC_50,_ ND – Not Determined.

*Structure Activity Relationship (SAR)*. Structure activity relationship study suggests that, substitution or replacement of phenyl ring has significant effect on anti-leishmanial activity as well as cytotoxicity of these analogues. Among these analogues, un-substituted phenyl derivative displayed significant inhibition of promastigotes, axenic amastigote and intracellular amastigotes of *L. donovani*. Ortho-para directing methyl group substitution on ortho and para position enhanced the potency against axenic amastigotes and intracellular amastigotes of *L. donovani* significantly, while on para substitution anti-promastigote activity altered marginally. Methoxy, chloro group substitution had strong impact on anti-leishmanial activity of these analogues, position of substitution is vital in their inhibition activity. Ortho substitution of methoxy group showed excellent increase in inhibition potency against promastigotes and both amastigote forms of *L. donovani*. While methoxy substitution on ortho position slightly increased the anti-leishmanial activity, meta substitution resulted in drastic decline in activity. Chloro substitution on para and meta position has significantly increased the inhibition potency against tested forms, while on ortho substitution has marginally affected the activity. Meta directing nitro and fluoro substitution on para position haven’t showed any considerable effect on inhibition potency of these analogues. However, fluoro substitution on ortho position and chloro di-substitution (ortho and meta) significantly decreased the anti-leishmanial potency. Replacement of phenyl ring with benzyl ring has significantly enhanced the activity. Even though pyridyl replacement resulted in increased anti-leishmanial activity, it also enhanced the cytotoxicity of these analogues. SAR studies suggested that, ortho-para directing methoxy group on para position, chloro group on para and meta position and benzyl replacement was recommended for anti-leishmanial activity of these analogues against *L. donovani*.

Through the present study, we have identified new lead molecules with potent anti-leishmaninal activity against *L. infantum*and *L. donovani.* Structure activity relationship studies would be utilised to optimize the lead molecules to generate clinically effective against both drug sensitive and resistance strains of *L. infantum*and *L. donovani.* Exact mechanism of action of these molecules for thier potential activity is needs to be evaluated. We hypothesize based upon literature reports, that these molecules may exhibit the observed potent activity through interaction with nucleic acids.

## 3 Conclusion

In summary, we have successfully applied molecular hybridization technique to design piperazinyl-β-carboline-3-carboxamide derivatives as anti-leishmanial agents. Designed molecules were synthesized and evaluated for anti-leishmanial activity against *L. infantum* and *L*. *donovani*. Analogues displayed significant inhibition of promastigote forms (EC_50_ < 20 μM) were further evaluated against amastigote forms of respective species. Most of these reported analogues displayed potent anti-leishmanial activity (promastigote and amastigote forms) against the both tested strains with better selectivity index. Compounds such as, **7d, 7g** and **7c** wereexhibited potent inhibition of promastigote (EC_50_ 1.59, 1.47 and 3.73 µM respectively) and amastigote (EC_50_ 1.4, 1.9 and 2.6 µM respectively) of *L. infantum* than standard drugs miltefosine, pentamidine. SAR studied suggested that, ortho-para directing methoxy, chloro groups substitution on para position as well as methyl group on ortho position favored the anti-leishmanial activity against *L. infantum*.

More interestingly, most of these compounds showed potent anti-leishmanial activity against *L. donovani*. Especially, compound **7d** displayed potent inhibition of *L. donovani* promastigotes, axenic amastigotes and intracellular amastigotes with EC_50_ 0.91, 0.9 and 1.3 µM respectively. Analogues **7n**, **7g** and **7h** were exhibited potent inhibition activity against all tested forms of *L. donovani*. SAR studies suggested that, ortho-para directing methoxy group on para position, chloro group on para and meta position were recommended for anti-leishmanial activity against *L. donovani*. Although, pyridyl replacement has increased the inhibition activity as well cytotoxicity and benzyl replacement has enhanced the anti-leishmanial activity with better selectivity.

## 4 Experimental protocols

### 4.1 Chemistry

All solvents and reagents purchased from Sigma or Merck companies are used as received without further purification. Solvent system used throughout the experimental work for running Thin Layer Chromatography (TLC) was ethyl acetate and hexane mixture (6:4) in order to monitor the reaction. Column chromatography was performed using silica gel (100–200 mesh, SRL, India) as stationary phase and mixture of ethyl acetate and hexane as mobile phase. Melting points are uncorrected and were determined in open capillary tubes on a Precision Buchi B530 (Flawil, Switzerland) melting point apparatus containing silicon oil. IR spectra of the synthesized compounds were recorded using FT-IR spectrophotometer (Shimadzu IR Prestige 21, India). ^1^H NMR spectra were recorded on a Bruker DPX-400 spectrometer (Bruker India Scientific Pvt. Ltd., Mumbai) using TMS as an internal standard (chemical shifts in d, ppm). Elemental analysis was performed on Vario EL III M/s Elementar C, H, N and S analyzer (ElementarAnalysensysteme GmbH, Germany). The ESMS were recorded on MICROMASS Quattro-II LCMS system (Waters Corporation, Milford, USA).

### 4.2 General procedure for the preparation of **7**

To a stirred solution of **(6)** (0.29 g, 0.001 mol) in dry THF, HOBt (0.16 g, 0.012 mol) and EDCI HCl (0.23 g, 0.012 mol) were added and continued stirring for 30 min. To the reaction mixture, substituted phenylpiperazine (0.001 mol) was added under ice cold temperature and the reaction mixture was further stirred at room temperature for 6 h. After completion of reaction as monitored by TLC, solvent was evaporated under vacuum. Reaction mixture was extracted with ethyl acetate (2 × 20 mL), collected organic layer was dried over Na_2_SO_4_ and concentrated under vacuum to get **(7a-p)**.

#### 4.2.1 (9-methyl-1-phenyl-9H-pyrido[3,4-*b*]indol-3-yl)(4-phenylpiperazin-1-yl)methanone (**7a**)

White solid; Yield 76%; mp 232–234 °C; R_f_ 0.45 (Hexane:EtOAc = 6:4); IR *ν*max/cm^−1^ (KBr): 3043, 1636, 1564, 1468; ^1^H NMR (400 MHz, CDCl_3_): *δ* 8.54 (s, 1H), 8.21 (d, *J* = 7.8 Hz, 1H), 7.70–7.61 (m, 3H), 7.58–7.44 (m, 4H), 7.36 (t, *J* = 7.2 Hz, 1H), 7.30–7.28 (m, 2H), 6.96–6.90 (m, 3H), 4.04 (brs, 4H), 3.52 (s, 3H), 3.33–3.23 (m, 4H); ESI-MS: *m*/*z* 447 ((M + 1), 100%); Anal. Calcd for C_29_H_26_N_4_O: C, 78.00; H, 5.87; N, 12.55, Found: C, 78.02; H, 5.85; N, 12.51.

#### 4.2.2 (9-methyl-1-phenyl-9H-pyrido[3,4-*b*]indol-3-yl)(4-p-tolylpiperazin-1-yl)methanone (**7b**)

White solid; Yield 72%; mp 188–190 °C; R_f_ 0.40 (Hexane:EtOAc = 6:4); IR *ν*max/cm^−1^ (KBr): 3059, 1652, 1558, 1448; ^1^H NMR (400 MHz, CDCl_3_): *δ* 8.55 (s, 1H), 8.23 (d, *J* = 7.8 Hz, 1H), 7.68–7.64 (m, 3H), 7.58–7.53 (m, 3H), 7.48 (d, *J* = 8.3 Hz, 1H), 7.38 (t, *J* = 7.5 Hz, 1H), 7.11 (d, *J* = 8.2 Hz, 2H), 6.88 (d, *J* = 8.5 Hz, 2H), 4.06–4.04 (m, 4H), 3.54 (s, 3H), 3.29–3.18 (m, 4H), 2.30 (s, 3H); ESI-MS *m*/*z* 461 ((M + 1), 100%); Anal. Calcd for C_30_H_28_N_4_O: C, 78.23; H, 6.13; N, 12.16, Found: C, 78.22; H, 6.16; N, 12.14.

#### 4.2.3 (9-methyl-1-phenyl-9H-pyrido[3,4-*b*]indol-3-yl)(4-o-tolylpiperazin-1-yl)methanone (**7c**)

White solid; Yield 64%; mp 148–150 °C; R_f_ 0.43 (Hexane:EtOAc = 6:4); IR *ν*max/cm^−1^ (KBr): 3053, 1636, 1552, 1419; ^1^H NMR (400 MHz, CDCl_3_): *δ* 8.51 (s, 1H), 8.21 (d, *J* = 7.7 Hz, 1H), 7.65–7.62 (m, 3H), 7.55–7.50 (m, 3H), 7.45 (d, *J* = 8.2 Hz, 1H), 7.36 (t, *J* = 7.5 Hz, 1H), 7.20–7.15 (m, 2H), 7.01–6.99 (m, 2H), 4.02–4.00 (m, 4H), 3.51 (s, 3H), 3.06–2.94 (m, 4H), 2.35 (s, 3H); ESI-MS *m*/*z* 461 ((M + 1), 100%); Anal. Calcd for C_30_H_28_N_4_O: C, 78.23; H, 6.13; N, 12.16, Found: C, 78.20; H, 6.12; N, 12.18.

#### 4.2.4 (4-(4-methoxyphenyl)piperazin-1-yl)(9-methyl-1-phenyl-9H-pyrido[3,4-*b*]indol-3-yl)methanone (**7d**)

White solid; Yield 82%; mp 170–172 °C; R_f_ 0.30 (Hexane:EtOAc = 6:4); IR *ν*max/cm^−1^ (KBr): 3064, 1651, 1516, 1454, 1255; ^1^H NMR (400 MHz, CDCl_3_): *δ* 8.53 (s, 1H), 8.21 (d, *J* = 7.8 Hz, 1H), 7.66–7.62 (m, 4H), 7.54–7.50 (m, 4H), 7.45 (d, *J* = 8.3 Hz, 1H), 7.36 (t, *J* = 7.5 Hz, 1H), 6.86 (d, *J* = 8.8 Hz, 2H), 4.10 (brs, 4H), 3.77 (s, 3H), 3.51 (s, 3H), 3.25–3.17(m, 4H); ESI-MS *m*/*z* 477 ((M + 1), 100%); Anal. Calcd for C_30_H_28_N_4_O_2_: C, 75.61; H, 5.92; N, 11.76, Found: C, 75.65; H, 5.88; N, 11.71.

#### 4.2.5 4.2.6.(4-(3-methoxyphenyl)piperazin-1-yl)(9-methyl-1-phenyl-9H-pyrido[3,4-*b*]indol-3-yl)methanone (**7e**)

White solid; Yield 74%; mp 196–198 °C; R_f_ 0.35 (Hexane:EtOAc = 6:4); IR *ν*max/cm^−1^ (KBr): 3057, 1625, 1556, 1454, 1435, 1253; ^1^H NMR (400 MHz, CDCl_3_): *δ* 8.54 (s, 1H), 8.21 (d, *J* = 7.8 Hz, 1H), 7.66–7.62 (m, 3H), 7.56–7.50 (m, 3H), 7.46 (d, *J* = 8.3 Hz, 1H), 7.36 (t, *J* = 7.5 Hz, 1H), 7.20 (t, *J* = 8.0 Hz, 1H), 6.62–6.48 (m, 3H), 4.13–4.07 (m, 4H), 3.80 (s, 3H), 3.52 (s, 3H), 3.36–3.26 (m, 4H); ESI-MS *m*/*z* 477 ((M + 1), 100%); Anal. Calcd for C_30_H_28_N_4_O_2_: C, 75.61; H, 5.92; N, 11.76, Found: C, 75.58; H, 5.94; N, 11.79.

#### 4.2.6 (4-(2-methoxyphenyl)piperazin-1-yl)(9-methyl-1-phenyl-9H-pyrido[3,4-*b*]indol-3-yl)methanone (**7f**)

White solid; Yield 62%; mp 168–170 °C; R_f_ 0.35 (Hexane:EtOAc = 6:4); IR *ν*max/cm^−1^ (KBr): 3053, 1643, 1556, 1496, 1240; ^1^H NMR (400 MHz, CDCl_3_): *δ* 8.54 (s, 1H), 8.23 (d, *J* = 7.8 Hz, 1H), 7.68–7.64 (m, 3H), 7.58–7.52 (m, 3H), 7.48 (d, *J* = 8.3 Hz, 1H), 7.38 (t, *J* = 7.5 Hz, 1H), 7.06–7.04 (m, 1H), 6.96–6.90 (m, 3H), 4.08 (brs, 4H), 3.90 (s, 3H), 3.54 (s, 3H), 3.22–3.12 (m, 4H); ESI-MS *m*/*z* 477 ((M + 1), 100%); Anal. Calcd for C_30_H_28_N_4_O_2_: C, 75.61; H, 5.92; N, 11.76, Found: C, 75.63; H, 5.97; N, 11.73.

#### 4.2.7 (4-(4-chlorophenyl)piperazin-1-yl)(9-methyl-1-phenyl-9H-pyrido[3,4-*b*]indol-3-yl)methanone (**7g**)

White solid; Yield 76%; mp 160–162 °C; R_f_ 0.40 (Hexane:EtOAc = 6:4); IR *ν*max/cm^−1^ (KBr): 3055, 1633, 1556, 1471, 756; ^1^H NMR (400 MHz, CDCl_3_): *δ* 8.57 (s, 1H), 8.24 (d, *J* = 7.8 Hz, 1H), 7.68–7.64 (m, 3H), 7.56–7.53 (m, 3H), 7.50–7.48 (m, 1H), 7.41–7.37 (m, 1H), 7.26 (d, *J* = 8.9 Hz, 2H), 7.02–6.95 (m, 2H), 4.10 (brs, 4H), 3.54 (s, 3H), 3.34–3.25 (m, 4H); ESI-MS *m*/*z* 481 ((M + 1), 100%), 483 ((M + 3), 33%); Anal. Calcd for C_29_H_25_ClN_4_O: C, 72.42; H, 5.24; N, 11.65, Found: C, 72.39; H, 5.26; N, 11.63.

#### 4.2.8 (4-(3-chlorophenyl)piperazin-1-yl)(9-methyl-1-phenyl-9H-pyrido[3,4-*b*]indol-3-yl)methanone (**7h**)

White solid; Yield 70%; mp 130–132 °C; R_f_ 0.42 (Hexane:EtOAc = 6:4); IR *ν*max/cm^−1^ (KBr): 3069, 1614, 1556, 1487, 736; ^1^H NMR (400 MHz, CDCl_3_): *δ* 8.52 (s, 1H), 8.19 (d, *J* = 7.9 Hz, 1H), 8.01–7.99 (m, 2H), 7.66–7.53 (m, 5H), 7.39–7.35 (m, 1H), 7.21 (t, *J* = 8.1 Hz, 1H), 6.93 (t, *J* = 2.1 Hz, 1H), 6.88–6.82 (m, 2H), 4.12–4.06 (m, 4H), 3.54 (s, 3H), 3.38–3.31 (m, 4H); ESI-MS *m*/*z* 481 ((M + 1), 100%), 483 ((M + 3), 33%); Anal. Calcd for C_29_H_25_ClN_4_O: C, 72.42; H, 5.24; N, 11.65, Found: C, 72.46; H, 5.27; N, 11.68.

#### 4.2.9 (4-(2-chlorophenyl)piperazin-1-yl)(9-methyl-1-phenyl-9H-pyrido[3,4-*b*]indol-3-yl)methanone (**7i**)

White solid; Yield 66%; mp 152–154 °C; R_f_ 0.40 (Hexane:EtOAc = 6:4); IR *ν*max/cm^−1^ (KBr): 3043, 1629, 1556, 1435, 734; ^1^H NMR (400 MHz, CDCl_3_): *δ* 8.51 (s, 1H), 8.21 (d, *J* = 7.8 Hz, 1H), 7.65–7.62 (m, 3H), 7.56–7.44 (m, 4H), 7.46–7.44 (m, 2H), 7.24–7.20 (m, 1H), 7.05–6.97 (m, 2H), 4.04 (brs, 4H), 3.51 (s, 3H), 3.19–3.08 (m, 4H);ESI-MS *m*/*z* 481 (((M + 1), 100%), 483 ((M + 3), 33%); Anal. Calcd for C_29_H_25_ClN_4_O: C, 72.42; H, 5.24; N, 11.65, Found: C, 72.40; H, 5.20; N, 11.64.

#### 4.2.10 (9-methyl-1-phenyl-9H-pyrido[3,4-*b*]indol-3-yl)(4-(4-nitrophenyl)piperazin-1-yl)methanone (**7j**)

Yellow solid; Yield 70%; mp 200–202 °C; R_f_ 0.30 (Hexane:EtOAc = 6:4); IR *ν*max/cm^−1^ (KBr): 3053, 1633, 1597, 1471, 1303, 754; ^1^H NMR (400 MHz, CDCl_3_): *δ* 8.55 (s, 1H), 8.19–8.14 (m, 3H), 7.97 (d, *J* = 7.1 Hz, 2H), 7.64–7.51 (m, 5H), 7.38–7.34 (m, 1H), 6.84 (d, *J* = 9.4 Hz, 2H), 4.19–4.06 (m, 4H), 3.51 (s, 3H), 3.64–3.56 (m, 4H);ESI-MS *m*/*z* 492 ((M + 1),100%); Anal. Calcd for C_29_H_25_N_5_O_3_: C, 70.86; H, 5.13; N, 14.25, Found: C, 70.82; H, 5.12; N, 14.28.

#### 4.2.11 (4-(4-fluorophenyl)piperazin-1-yl)(9-methyl-1-phenyl-9H-pyrido[3,4-*b*]indol-3-yl)methanone (**7k**)

White solid; Yield 76%; mp 188–190 °C; R_f_ 0.40 (Hexane:EtOAc = 6:4); IR *ν*max/cm^−1^ (KBr): 3057, 1614, 1556, 1487, 736; ^1^H NMR (400 MHz, CDCl_3_): *δ* 8.56 (s, 1H), 8.23 (d, *J* = 7.7 Hz, 1H), 7.68–7.65 (m, 3H), 7.58–7.53 (m, 3H), 7.48 (d, *J* = 8.4 Hz, 1H), 7.38 (td, *J* = 7.6, 0.8 Hz, 1H), 7.06–6.96 (m, 2H), 6.96–6.88 (m, 2H), 4.06–4.05 (m, 4H), 3.54 (s, 3H), 3.26–3.16 (m, 4H);ESI-MS *m*/*z* 465 ((M + 1),100%); Anal. Calcd for C_29_H_25_FN_4_O: C, 74.98; H, 5.42; N, 12.06, Found: C, 74.94; H, 5.45; N, 12.03.

#### 4.2.12 (4-(2-fluorophenyl)piperazin-1-yl)(9-methyl-1-phenyl-9H-pyrido[3,4-*b*]indol-3-yl)methanone (**7l**)

White solid; Yield 68%; mp 168–170 °C; R_f_ 0.45 (Hexane:EtOAc = 6:4); IR *ν*max/cm^−1^ (KBr): 3055, 1633, 1556, 1471, 1435, 756; ^1^H NMR (400 MHz, CDCl_3_): *δ* 8.53 (s, 1H), 8.21 (d, *J* = 7.8 Hz, 1H), 7.65–7.62 (m, 3H), 7.55–7.50 (m, 3H), 7.45 (d, *J* = 8.3 Hz, 1H), 7.36 (t, *J* = 7.5 Hz, 1H), 7.07–6.93 (m, 4H), 4.05–4.04 (m, 4H), 3.51 (s, 3H), 3.23–3.13 (m, 4H); ESI-MS *m*/*z* 465 ((M + 1),100%); Anal. Calcd for C_29_H_25_FN_4_O: C, 74.98; H, 5.42; N, 12.06, Found: C, 74.96; H, 5.39; N, 12.10.

#### 4.2.13 (4-(2,3-dichlorophenyl)piperazin-1-yl)(9-methyl-1-phenyl-9H-pyrido[3,4-*b*]indol-3-yl)methanone (**7m**)

White solid; Yield 74%; mp 166–168 °C; R_f_ 0.32 (Hexane:EtOAc = 6:4); IR *ν*max/cm^−1^ (KBr): 3059, 1614, 1556, 1438, 790, 736; ^1^H NMR (400 MHz, CDCl_3_): *δ* 8.52 (s, 1H), 8.21 (d, *J* = 7.8 Hz, 1H), 7.67–7.62 (m, 3H), 7.58–7.48 (m, 3H), 7.46 (d, *J* = 8.3 Hz, 1H), 7.36 (t, *J* = 7.5 Hz, 1H), 7.20–7.11 (m, 2H), 6.95 (dd, *J* = 7.6, 1.9 Hz, 1H), 4.04 (brs, 4H), 3.51 (s, 3H), 3.18–3.08 (m, 4H); ESI-MS *m*/*z* 516 (((M + 1), 100%), 518 ((M + 3), 66%), 518 ((M + 5), 10%)); Anal. Calcd for C_29_H_24_Cl_2_N_4_O: C, 67.58; H, 4.69; N, 10.87, Found: C,67.62; H, 4.64; N, 10.91.

#### 4.2.14 (4-benzylpiperazin-1-yl)(9-methyl-1-phenyl-9H-pyrido[3,4-*b*]indol-3-yl)methanone (**7n**)

White solid; Yield 78%; mp 158–160 °C; R_f_ 0.40 (Hexane:EtOAc = 6:4); IR *ν*max/cm^−1^ (KBr): 3055, 1614, 1552, 1438, 1409; ^1^H NMR (400 MHz, CDCl_3_): *δ* 8.50 (s, 1H), 8.22 (d, *J* = 7.7 Hz, 1H), 7.66–7.62 (m, 3H), 7.56–7.52 (m, 3H), 7.47 (d, *J* = 8.3 Hz, 1H), 7.39–7.34 (m, 5H), 7.30–7.28 (m, 1H), 3.91–3.87 (m, 4H), 3.57 (s, 2H), 3.52 (s, 3H), 2.62–2.48 (m, 4H); ESI-MS *m*/*z* 461 ((M + 1), 100%); Anal. Calcd for C_30_H_28_N_4_O: C, 78.23; H, 6.13; N, 12.16, Found: C, 78.26; H, 6.11; N, 12.19.

#### 4.2.15 (9-methyl-1-phenyl-9H-pyrido[3,4-*b*]indol-3-yl)(4-(pyridin-4-yl)piperazin-1-yl)methanone (**7o**)

Yellowish white solid; Yield 68%; mp 198–200 °C; R_f_ 0.35 (Hexane:EtOAc = 2:8); IR *ν*max/cm^−1^ (KBr): 3053, 1620, 1558, 1456; ^1^H NMR (400 MHz, CDCl_3_): *δ* 8.59 (s, 1H), 8.31 (d, *J* = 5.4 Hz, 2H), 8.24 (d, *J* = 7.8 Hz, 1H), 7.68–7.62 (m, 3H), 7.58–7.55 (m, 3H), 7.50 (d, *J* = 8.4 Hz, 1H), 7.38 (t, *J* = 7.5 Hz, 1H), 6.68 (d, *J* = 6.0 Hz, 2H), 4.10–4.03 (m, 4H), 3.51 (s, 3H), 3.53–3.42 (m, 4H);ESI-MS *m*/*z* 448 ((M + 1), 100%); Anal. Calcd for C_28_H_25_N_5_O: C, 75.15; H, 5.63; N, 15.65, Found: C, 75.19; H, 5.68; N, 15.68.

#### 4.2.16 (9-methyl-1-phenyl-9H-pyrido[3,4-*b*]indol-3-yl)(4-(pyridin-2-yl)piperazin-1-yl)methanone (**7p**)

Yellowish white solid; Yield 62%; mp 174–176 °C; R_f_ 0.30 (Hexane:EtOAc = 2:8); IR *ν*max/cm^−1^ (KBr): 3053, 1614, 1556, 1487, 1471; ^1^H NMR (400 MHz, CDCl_3_): *δ* 8.57 (s, 1H), 8.24–8.21 (m, 2H), 7.69–7.65 (m, 3H), 7.58–7.47 (m, 5H), 7.38 (t, *J* = 7.5 Hz, 1H), 6.69–6.66 (m, 2H), 4.02 (brs, 4H), 3.71–3.69 (m, 4H), 3.55 (s, 3H); ESI-MS *m*/*z* 448 ((M + 1), 100%); Anal. Calcd for C_28_H_25_N_5_O: C, 75.15; H, 5.63; N, 15.65, Found: C, 75.12; H, 5.61; N, 15.62.

### 4.3 Biological evaluation

#### 4.3.1 Cytotoxicity assay

HeLa cell cytotoxicity studies were carried out as described previously. Briefly, the cells were cultured in DMEM supplemented with 10% fetal calf serum and 2 mM l-glutamine. Cells were plated at initial cell concentration of 2 × 10^4^ cells/well and incubated with the compounds for ∼65 h prior to addition of Alamar Blue solution for further 5 h [36].

#### 4.3.2 Promastigote assay

*L. donovani*promastigotes were cultured at 37 °C in M199 medium supplemented with 10% heat-inactivated fetal calf serum. *L. infantum* JPCM5 MCAN/ES/98/LLM-87 promastigotes were cultured at 26 °C in modified Eagle’s medium (HOMEM) [37]. Parasites were incubated with serial dilutions of compounds for 72 h, followed by Alamar blue-based assay as previously described [38].

#### 4.3.3 Axenic amastigote assay

*L. donovani*LdBOB axenic amastigotes or *L. infantum* JPCM5 MCAN/ES/98/LLM-87 were cultured at 37 °C in modified Eagle’s medium (HOMEM; *L. infantum*) and axenic amastigotes were incubated for 72 h with compounds, followed by Alamar blue-based assay as previously described [38].

#### 4.3.4 Intra macrophage *L. donovani* (amastigote) assay

THP-1 (human monocytic leukemia) cells were a kind gift from Dr Susan Wylie (Dundee) and maintained in minimal essential medium plus 10% (vol/vol) FBS. An intracellular *Leishmania* assay using LdBOB amastigotes were performed utilizing PMA differentiated THP-1 cells infected overnight with axenic amastigotes, prior to compounds being added and incubated for further 72 h, subsequent microscopy-based readout was used to determine EC_50_ values [38]. Briefly, THP-1 cells (20,000 per well, 200 µL) were plated into 96 well plates in presence of 10 nM PMA and incubated at 37 °C in 5% CO_2_ atmosphere for 75 h. The adhered cells were then washed with phosphate buffered saline supplemented with 1 mM CaCl_2_, 0.5 mM MgCl_2_, 0.1% (w/v) bovine serum albumin and using LdBOB amastigotes added to all wells at a multiplicity of infection of 5 (100,000 amastigotes per well) and incubated for 18 h. Any remaining extracellular amastigotes were removed and the adhered cells were washed with the same supplemented PBS solution as above. Pre-aliquoted compounds in a serial dilution were added from another plate, such that the total well volume was 200 μL and incubated for further 72 h. Cells were fixed with 100% methanol, stained with Giemsa and examined microscopically. Number of intracellular amastigotes (∼200 macrophages per well) were determined and the percentage infection was established compared to an untreated control (100%) allowing EC_50_ values to be calculated [38].

## Conflicts of interest

There are no conflicts of interest declared by the authors.
